# Construction and Validation of the Identity Crisis and Histrionic Personality Disorder Scale for Young Adult Girls

**DOI:** 10.7759/cureus.85822

**Published:** 2025-06-12

**Authors:** Rajeev Misra, Monika Agarwal, Divyanshi Singh, Akanksha Mishra, Nishi Rastogi, Hansika Singhal, Raj Gopal Reddy

**Affiliations:** 1 Community Medicine and Public Health, King George's Medical University, Lucknow, IND; 2 Cardiovascular Thoracic Surgery, King George's Medical University, Lucknow, IND; 3 Statistics, National Post Graduate College, Lucknow, IND; 4 Psychology, Lucknow University, Lucknow, IND; 5 Clinical Operations, Chandan Hospital, Lucknow, IND

**Keywords:** histrionic personality disorder, identity crisis, reliability, scale development, validity

## Abstract

Background

This study centers on the development and validation of a psychometric scale designed to assess identity crisis and histrionic personality disorder (HPD), addressing a significant gap in current diagnostic tools. Identity crises, often characterized by confusion about one’s self-concept and societal roles, commonly emerge during adolescence and can lead to maladaptive behaviors. HPD, marked by attention-seeking and emotional dysregulation, shares notable overlaps with identity crises, particularly in disrupted self-concept and a heightened dependence on external validation.

Methods

A multiphase process guided the scale’s development, including a comprehensive literature review, expert evaluations, and pilot testing. Initially, 38 items were generated based on core theoretical constructs of identity crisis and HPD. Following expert review, the scale was refined to 28 items and pilot-tested on a diverse sample of 275 participants aged 18-25.

Results

Exploratory factor analysis identified five core dimensions: identity instability, dramatic expression, excessive concern with physical appearance, impulsivity, and relational intimacy. Confirmatory factor analysis further supported the model’s robustness, yielding strong fit indices (Comparative Fit Index = 0.918, Root Mean Square Error of Approximation = 0.045).

Conclusions

The scale demonstrated excellent internal consistency (Cronbach’s α = 0.94) and strong construct validity. Its multidimensional framework holds substantial value for both clinicians and researchers in assessing identity-related disturbances and HPD traits. While developed for young adults, future research should explore its applicability across broader populations and cultural contexts. This tool effectively bridges theoretical understanding with practical application, offering a foundation for targeted interventions and advancing the study of identity and personality disorders.

## Introduction

Identity development is a fundamental aspect of psychological growth, with adolescence and early adulthood serving as critical periods for the formation of self-concept. Erikson’s psychosocial theory posits that identity consolidation involves navigating the dual challenges of internal self-definition and external social expectations [1]. Successfully resolving these challenges leads to a coherent and stable sense of self, whereas unresolved conflicts may result in an identity crisis. Such crises often manifest as confusion regarding personal roles, values, and aspirations, which can negatively impact emotional regulation, interpersonal relationships, and overall life satisfaction [2].

Adolescents are particularly vulnerable to identity disturbances due to the confluence of biological, psychological, and social transitions they undergo during this stage. External pressures, including parental expectations, peer comparisons, and societal ideals reinforced by social media, further complicate the identity formation process [3]. Research has shown that prolonged exposure to these pressures can foster chronic self-doubt and maladaptive coping strategies, such as a reliance on external validation or impulsive behaviors [4].

Histrionic personality disorder (HPD), classified under Cluster B personality disorders in the Diagnostic and Statistical Manual of Mental Disorders, Fifth Edition (DSM-5) [5], represents a maladaptive pattern characterized by emotional dysregulation and attention-seeking behavior. Key features of HPD include exaggerated emotional expression, superficial interpersonal connections, and a pervasive need for approval. These overt traits often conceal deeper difficulties related to self-esteem and identity coherence [6]. For example, individuals with HPD may engage in dramatic or provocative behaviors as compensatory mechanisms to mask feelings of inadequacy or insecurity.

The intersection between identity crises and HPD has received growing attention in psychological literature. Both involve disruptions in self-concept, although identity crises are typically developmental and transient; HPD traits can become chronic if left unaddressed [7]. An adolescent struggling with identity instability may, for instance, resort to histrionic behaviors such as exaggerating achievements or forming overly intense relationships in an attempt to compensate for an unclear sense of self. Over time, these behavioral patterns can solidify, potentially contributing to the development of HPD.

Despite the theoretical overlap, empirical research exploring the interplay between identity crises and HPD remains limited. Most existing diagnostic tools assess these constructs separately, often failing to account for their interconnections. This gap highlights the need for a comprehensive instrument capable of capturing the nuanced relationship between identity instability and HPD traits. Such a tool would allow clinicians and researchers to better understand, assess, and intervene in cases where identity-related disturbances contribute to personality pathology.

Grounded in established theories of identity development, personality disorders, and psychometric evaluation, the present study aims to fill this gap. By developing and validating a novel scale, this research seeks to provide a reliable, multidimensional instrument that integrates theoretical frameworks with clinical applicability. Ultimately, this scale is intended to enhance diagnostic accuracy, support targeted interventions, and contribute to a deeper understanding of identity-related phenomena in both clinical and research settings.

DSM-5-TR personality disorder clusters

Personality disorders are categorized by the DSM-5-TR into three clusters - A, B, and C - each encompassing a distinct group of disorders characterized by specific symptoms, behavioral tendencies, and underlying psychological patterns [8,9].

Cluster A includes personality disorders marked by odd or eccentric behaviors. This cluster comprises schizoid, schizotypal, and paranoid personality disorders. Individuals within this group often display social detachment, pervasive distrust, and difficulties forming close relationships [10].

Cluster B encompasses disorders associated with dramatic, emotional, or erratic behavior. This includes antisocial, borderline, narcissistic, and HPDs. Individuals in this cluster commonly exhibit impulsivity, emotional instability, and challenges in sustaining healthy interpersonal connections [11].

Cluster C involves personality disorders characterized by anxiety and fear. These include avoidant, dependent, and obsessive-compulsive personality disorders. People in this cluster often struggle with chronic anxiety, fear of abandonment, and an excessive need for control or perfectionism [12].

Personality is defined as a stable set of behavioral patterns through which individuals perceive, interpret, and engage with the world. A personality disorder arises when these patterns become rigid and maladaptive, significantly impairing social or occupational functioning and causing distress in interpersonal relationships [13].

Yalch et al. [14] provide evidence that adverse childhood experiences, particularly child maltreatment and sexual abuse, are significant risk factors for the development of HPD. Both identity crises and HPD traits represent important public health concerns due to their association with long-term psychological difficulties and reduced quality of life.

## Materials and methods

Scale development process

Conceptual Framework Development

The foundation of the scale was established through a comprehensive review of theoretical and empirical literature. Key constructs were drawn from Erikson’s identity development framework [1], which emphasizes the dynamic interplay between self-perception and social roles. Contemporary models of personality disorders, including Millon et al.’s biopsychosocial approach [6], further informed the identification of dimensions relevant to HPD.

Expert Panel

An expert panel comprising five clinical psychologists and psychiatrists with expertise in identity development and personality disorders was assembled to evaluate the item pool. Items that received a content validity rating below 3 on a 4-point Likert scale were either excluded or revised to ensure clarity, relevance, and clinical utility.

Core Dimensions

The scale was structured to capture several psychological and behavioral dimensions associated with identity disturbance and histrionic traits. Self-concept confusion reflects uncertainty about one’s personal values, roles, and overall identity, which often results in a fragmented sense of self. Emotional instability refers to rapid and unpredictable shifts in emotional states, frequently accompanied by impulsive reactions. Attention-seeking behaviors involve an excessive reliance on external validation and approval to maintain a sense of self-worth. Dramatic expression highlights the use of exaggerated gestures, expressions, or speech to draw attention and communicate emotions intensely. Relational intimacy examines the individual’s perception of closeness in relationships and a strong dependency on interpersonal connections for emotional stability. Together, these dimensions offer a comprehensive framework for understanding the multifaceted nature of identity crises and histrionic personality tendencies.

Item generation

A pool of 38 items was initially developed to represent the identified dimensions. Each item was designed to align with the theoretical constructs, ensuring both content relevance and clarity. Example items included questions such as, “Do you frequently feel unsure of who you are?” and “Do you find yourself acting dramatically to capture others’ attention?”

Expert validation

The preliminary 38-item scale was reviewed by the expert panel. Using a 4-point Likert scale, each item was assessed for clarity, relevance, and representativeness. Based on their feedback, items deemed redundant or ambiguous were revised or removed. This process resulted in a refined 28-item scale, with the retained items demonstrating strong alignment with theoretical foundations and clinical observations.

Pilot testing

The refined scale was pilot-tested on a sample of 275 participants aged 18-25 years. This sample size was considered sufficient to ensure robust statistical analysis and reliable factor extraction, consistent with established guidelines for psychometric scale development. Internal consistency was evaluated using Cronbach’s alpha, while exploratory factor analysis (EFA) was conducted to uncover the underlying factor structure. Participants also provided qualitative feedback regarding item clarity and ease of understanding, which informed minor revisions to enhance comprehensibility.

Sampling and data collection

Participants were recruited through random sampling methods from universities, community centers, and online platforms. Data collection targeted young adult females aged 18-25 years from the state of Uttar Pradesh. Informed consent was obtained from all participants prior to their inclusion in the study. To ensure ethical compliance, all responses were anonymized, and confidentiality was maintained throughout the data collection process.

Inclusion Criteria

Participants were required to be aged between 18 and 25 years and to provide informed consent to participate in the study.

Exclusion Criteria

Individuals with a history of major psychiatric disorders such as schizophrenia or bipolar disorder were excluded. Additional exclusion criteria included current or recent (within the past six months) substance dependence or abuse, as well as the presence of neurological impairments such as dementia, epilepsy, or brain injury that could affect cognitive or emotional functioning.

Data analysis for scale validation

Internal consistency was assessed using Cronbach’s alpha, with values of 0.70 or higher indicating acceptable reliability. Item-total correlations were also computed to identify poorly performing items that could compromise the overall coherence of the scale. To explore the underlying factor structure, an EFA was conducted using principal axis factoring with varimax rotation. Factors were retained based on the criterion of eigenvalues greater than one, supported by visual inspection of the scree plot. To validate the factor structure identified through EFA, a confirmatory factor analysis (CFA) was subsequently conducted. Model fit was evaluated using multiple indices, including the Comparative Fit Index (CFI) and Tucker-Lewis Index (TLI), with values equal to or greater than 0.90 indicating good fit. Additional indices included the Root Mean Square Error of Approximation (RMSEA ≤0.06) and the Standardized Root Mean Square Residual (≤0.08), which further assessed the adequacy and robustness of the proposed model.

## Results

The scale was developed on a solid theoretical foundation, incorporating Erikson’s identity theory, Bowlby’s attachment theory, and Millon’s biopsychosocial model. An initial pool of 38 items was created to represent five proposed dimensions: self-concept confusion, emotional instability, attention-seeking, dramatic expression, and relational intimacy. After review by a panel of five psychologists with expertise in identity development and personality disorders, the scale was refined to 28 items. A pilot test was conducted with a sample of 275 adolescent girls from Uttar Pradesh, and minor adjustments were made based on participant feedback (Table 1).

**Table 1:** Demographics of the sample

Variable	Category	Frequency (n)	Percentage (%)
Age group	18-21 years	145	52.70%
	22-25 years	130	47.30%
Education level	Undergraduate	180	65.50%
	Postgraduate	95	34.50%
Location	Urban	158	57.50%
	Semi-urban/rural	117	42.50%
Language proficiency	Fluent in English	275	100%

The final version of the scale utilized a 5-point Likert format, ranging from 1 (Strongly Disagree) to 5 (Strongly Agree). Psychometric analysis revealed five distinct dimensions: identity instability, dramatic expression, appearance concerns, impulsivity, and relational intimacy. Reliability analysis indicated excellent internal consistency, with a Cronbach’s alpha of 0.94. EFA showed that these five factors accounted for a total of 72% of the variance: identity instability (21%), dramatic expression (18%), appearance concerns (14%), impulsivity (10%), and relational intimacy (9%). CFA further validated the structure, yielding favorable fit indices (CFI = 0.918, RMSEA = 0.045, TLI > 0.90).

Descriptive statistics indicated that subscale mean scores ranged from 3.8 to 4.2, with identity instability (M = 4.2, SD = 0.7) showing the highest mean and appearance concerns (M = 3.8, SD = 0.6) the lowest (Table 2). Correlational analyses demonstrated strong convergent validity. Specifically, the scale showed a significant negative correlation with the Self-Concept Clarity Scale (r = -0.71, p < 0.01) and positive correlations with the Impulsivity Scale (r = 0.64, p < 0.01) and the UCLA Loneliness Scale (r = -0.58, p < 0.01). These findings support the scale’s reliability and validity in assessing emotional and identity-related difficulties among adolescent girls.

**Table 2:** Descriptive statistics

Subscale	Mean score	SD	Range (min-max)
Identity instability	4.2	0.7	2.1-5.0
Dramatic expression	4	0.8	1.8-5.0
Appearance concerns	3.8	0.6	2.0-5.0
Impulsivity	3.9	0.7	2.3-5.0
Relational intimacy	4.1	0.7	2.0-5.0

Reliability analysis

The scale demonstrated excellent internal consistency, with a Cronbach’s alpha of 0.94, surpassing the standard threshold commonly used in psychometric evaluations. This high level of reliability highlights the scale’s strength and stability, reinforcing its suitability for both clinical use and research settings (Table 3).

**Table 3:** Reliability and validity analysis CFA, confirmatory factor analysis; CFI, Comparative Fit Index; EFA, exploratory factor analysis; RMSEA, Root Mean Square Error of Approximation; TLI, Tucker-Lewis Index

Analysis type	Result/value
Cronbach’s alpha	0.94, indicating excellent internal consistency
Total variance explained (EFA)	72%
Model fit indices (CFA)	CFI = 0.918, RMSEA = 0.045, TLI > 0.90
Convergent validity	Significant correlations with validated measures (e.g., r = 0.71 for identity clarity)

EFA

EFA was conducted to uncover the underlying structure of the newly developed scale, resulting in the extraction of five distinct and theoretically grounded factors. The first factor, identity instability, captured fluctuations in self-concept, inconsistencies in personal values, and a general lack of coherent identity. The second factor, dramatic expression, reflected exaggerated emotional displays and persistent attention-seeking behaviors, closely aligning with traits associated with HPD. The third factor, appearance concerns, indicated a heightened focus on physical appearance and the strategic use of appearance to influence how others perceive them. The fourth factor, impulsivity, measured the frequency and intensity of emotionally driven, spontaneous actions that often lack foresight or consideration of consequences. The fifth and final factor, relational intimacy, assessed difficulties in forming and maintaining deep, meaningful relationships, often characterized by fears of abandonment or superficial connections. Collectively, these five dimensions provide a comprehensive understanding of the emotional, behavioral, and interpersonal disruptions linked to identity crises and HPD (Table 4).

**Table 4:** EFA results EFA, exploratory factor analysis

Factor	Sample items (examples)	Variance (%)
Identity instability	“I often feel unsure of who I am.”	21%
Dramatic expression	“I find myself behaving dramatically to attract attention.”	18%
Appearance concerns	“I worry excessively about how others see my appearance.”	14%
Impulsivity	“I act on urges without thinking of consequences.”	10%
Relational intimacy	“I struggle to maintain stable, close relationships.”	9%
Total		72%

The factors collectively accounted for 72% of the total variance, indicating strong explanatory power for the scale’s underlying structure.

Qualitative Feedback

Participants offered qualitative feedback on the clarity and comprehensibility of the items. Minor revisions were implemented to enhance phrasing and reduce ambiguity, thereby improving the scale’s accessibility and usability.

Descriptive Statistics

Mean scores for each factor ranged from 3.8 to 4.2 on a 5-point Likert scale, suggesting a moderate to high prevalence of the measured constructs within the sample. Standard deviations ranged from 0.6 to 0.8, indicating a relatively consistent distribution of responses.

Group Differences

An analysis of demographic variables revealed no significant differences in scale scores based on gender. However, slight variations were observed across age groups: participants aged 18-21 scored marginally higher on the impulsivity and dramatic expression factors compared to those aged 22-25.

Correlations with external measures

The dimensions of the scale exhibited strong convergent and discriminant validity, as demonstrated by significant correlations with established psychological instruments. The identity instability dimension showed a strong negative correlation with self-concept clarity (r = -0.71, p < 0.01), indicating that individuals experiencing higher identity instability tend to have a less defined and consistent sense of self. The impulsivity factor demonstrated a moderate positive correlation with validated impulsivity scales (r = 0.64, p < 0.01), supporting its effectiveness in capturing emotionally driven, spontaneous behaviors. Meanwhile, the relational intimacy factor was negatively correlated with loneliness measures (r = -0.58, p < 0.01), suggesting that those struggling with intimacy often experience greater emotional loneliness and social disconnection. Collectively, these results affirm the scale’s psychometric strength and its capacity to assess key traits related to identity disturbance and personality dysfunction (Table 5).

**Table 5:** Correlations with external measures

Construct	Correlation coefficient (r)	Significance (p)
Self-concept clarity	-0.71	<0.01
Impulsivity Scale	0.64	<0.01
UCLA Loneliness Scale	-0.58	<0.01

## Discussion

The validated scale addresses a significant gap in existing psychometric tools by integrating the constructs of identity crisis and HPD into a comprehensive, multidimensional framework. Demonstrating high internal consistency (Cronbach’s alpha = 0.94), the scale proves to be a reliable instrument suitable for both clinical and research settings. EFA revealed five distinct dimensions - identity instability, dramatic expression, appearance concerns, impulsivity, and relational intimacy - highlighting the multifaceted nature of identity and personality disturbances. This empirical structure enhances the scale’s utility by capturing the complexity of emotional and behavioral patterns associated with identity-related disorders.

Clinically, the scale offers substantial value by providing nuanced insights that support the development of targeted, dimension-specific interventions. Its application allows mental health professionals to better understand the emotional and cognitive challenges individuals face, particularly in the context of identity confusion and attention-seeking behaviors. By identifying and addressing each dimension, practitioners can design interventions aimed at enhancing self-awareness, emotional regulation, and behavioral control. Notably, the relational intimacy component creates opportunities for therapeutic work focused on cultivating healthier and more stable interpersonal relationships. Overall, this tool serves as a critical bridge between theoretical constructs and real-world applications, enabling more precise diagnosis and effective treatment planning for individuals experiencing identity crises or exhibiting traits of HPD.

This study successfully fills a gap in psychological assessment by developing and validating a multidimensional scale that integrates identity crisis and HPD traits - two constructs that frequently co-occur but remain underexplored in unified diagnostic tools. The scale’s robust psychometric properties, including high internal consistency (α = 0.94), a clear factorial structure, and strong model fit indices, underscore its reliability and validity for both research and clinical contexts.

The five-factor structure aligns with theoretical foundations and clinical observations. For example, Erikson’s theory suggests that unresolved identity crises in adolescence can manifest in adulthood as emotional instability and confusion, which is reflected in the “Identity instability” and “Impulsivity” subscales. Similarly, the “Dramatic expression” and “Appearance concerns” dimensions correspond with DSM-5 descriptors of HPD, particularly the focus on exaggerated emotionality and appearance-based validation-seeking behaviors [5].

The observed correlations further support the scale’s construct validity. A strong inverse relationship with self-concept clarity (r = -0.71) aligns with prior findings suggesting that identity disturbance often precedes maladaptive personality features [2]. Despite its strengths, the study has several limitations. The sample, composed exclusively of young adult females, limits the generalizability of the findings across genders and age groups. Additionally, the cultural context of Uttar Pradesh may influence patterns of identity and behavioral expression, highlighting the need for cross-cultural validation. Prior research underscores the sociocultural roots of HPD symptoms, noting that emotional expression and interpersonal norms vary significantly across regions [9]. Likewise, the association between impulsivity traits and cluster B personality disorders, including HPD, has been consistently reported in psychiatric literature [11].

Limitations

This study presents several limitations that warrant acknowledgment. A primary constraint lies in the demographic composition of the sample, which consisted predominantly of young adults aged 18-25, with a higher proportion of female participants. This homogeneity may limit the generalizability of the findings to broader populations, including different age groups and gender identities. Additionally, cultural factors were not thoroughly examined, as the sample was drawn from a specific geographic and cultural setting. Considering the potential impact of sociocultural norms on identity development and personality expression, cross-cultural validation is essential to establish the scale’s applicability across diverse populations.

Future directions

Future research should aim to extend the scale’s validation to more diverse demographic groups, incorporating a broader range of ages, genders, and cultural backgrounds. Longitudinal studies could further strengthen the evidence base by assessing the scale’s predictive validity, particularly in tracking therapeutic outcomes and long-term psychological changes. Furthermore, exploring the scale’s use in nonclinical settings, such as educational institutions or organizational environments, may help identify subclinical traits and support early intervention strategies. These efforts would enhance the scale’s practical utility and reinforce its relevance in both clinical and psychosocial assessment contexts.

## Conclusions

This study introduces a validated psychometric scale for assessing identity crises and HPD traits, supported by strong psychometric properties and theoretical grounding. By addressing key limitations in existing assessment tools, the scale contributes to more accurate diagnosis, targeted therapeutic approaches, and a deeper understanding of identity-related disturbances. Continued research on its use across diverse populations and settings will further advance the field’s understanding of identity and personality pathology.

## Appendices

**Table 6:** Identity Crisis and Histrionic Personality Disorder Scale

Dimension	Strongly Disagree	Disagree	Neutral	Agree	Strongly Agree
Identity instability					
1. I feel unsure about who I am or what my purpose in life is.	1	2	3	4	5
2. I often feel like I am pretending to be someone I am not.	1	2	3	4	5
3. I frequently feel confused about my role or purpose in life.	1	2	3	4	5
4. I struggle to define my own values and goals independently of others’ expectations.	1	2	3	4	5
5. I frequently adopt new interests, hobbies, or styles based on people I want to impress.	1	2	3	4	5
6. I feel that seeking attention or approval from others helps define my identity.	1	2	3	4	5
Dramatic expression					
7. I often seek reassurance or approval from others.	1	2	3	4	5
8. I have been told that my emotions seem exaggerated or out of proportion.	1	2	3	4	5
9. I tend to use dramatic gestures or facial expressions to emphasize my points.	1	2	3	4	5
10. I use emotional expressions (e.g., crying or laughing) to gain sympathy or attention.	1	2	3	4	5
11. I talk loudly or interrupt others to get noticed.	1	2	3	4	5
12. I use phrases like “It was amazing” or “It was terrible” without providing specific examples.	1	2	3	4	5
Appearance concerns					
13. I believe my physical appearance is crucial in how others perceive me.	1	2	3	4	5
14. I feel anxious if I believe my appearance is not up to my standards.	1	2	3	4	5
15. I am preoccupied with achieving a particular standard of physical attractiveness.	1	2	3	4	5
16. I believe my appearance is a key factor in my success or happiness.	1	2	3	4	5
Impulsivity					
17. I find it difficult to resist immediate temptations or urges.	1	2	3	4	5
18. I make quick decisions without considering the consequences when I feel overjoyed.	1	2	3	4	5
19. I emphasize my feelings and opinions more than facts when discussing topics.	1	2	3	4	5
20. I behave impulsively when I am emotionally overwhelmed.	1	2	3	4	5
Relational intimacy					
21. I feel that I have a close, intimate connection with someone after only a brief interaction.	1	2	3	4	5
22. I often think that casual acquaintances or colleagues are my close friends.	1	2	3	4	5
23. I frequently tell others that I feel very close to them.	1	2	3	4	5
24. I find it challenging to maintain deep or meaningful connections with others.	1	2	3	4	5
25. When I am not the center of attention, I feel a sense of emptiness.	1	2	3	4	5
26. I boast about my accomplishments to gain admiration.	1	2	3	4	5
27. I dress or behave in ways that draw attention to myself.	1	2	3	4	5
28. I act flirtatiously with people I’ve just met to get noticed.	1	2	3	4	5
